# Mr. Gilbert and His Perpendicular Extraction

**Published:** 1852-01

**Authors:** George Brown

**Affiliations:** Lectr. Vety. Medl. Royal Agrictr. College, Cireucester.


### ARTICLE VI.

### *Mr. Gilbert and his Perpendicular Extraction.*

MESSRS. EDITORS:

*Gentlemen*—Being in London during the last month, I took an early opportunity of calling on Mr. Gilbert, surgeon dentist, of Suffolk street, for the purpose of requesting permission to examine his invention for the extraction of teeth; I have to acknowledge myself indebted to his courtesy for the trouble he took to explain to me the construction of his instrument, with his method of using it in various cases.

At first sight, I was impressed with the very ingenious nature of the invention, and subsequent consideration has left that impression uneffaced; but, that its advantage renders it pre-eminently superior to all other modes of operating, I am disposed to doubt.

A perusal of Mr. Gilbert's work will clearly convince the reader that the professed object of the fulcrum apparatus is to allow of the tooth being removed in a direction perpendicular to the axis; Mr. Gilbert's words are sufficiently definite, when he states his perfect concurrence in the expressed opinion of John Hunter, that "it would be best to attempt the extraction of the tooth in a direction perpendicular to its axis, and not by means of the *lateral* motion required by the forceps," and further goes on to remark that he succeeded at last in attaining the object sought by the invention of an apparatus which should afford a fulcrum external to the mouth—his success is attested by testimonials both from patients, who have placed themselves under his care, and from several scientific men who have inspected or used the instrument. The elevation of the tooth by *one movement* only, is more than once asserted; the escape from the "painful wriggle of the forceps," in the ordinary method of extraction, is triumphantly set forth; and as a climax, in the last communication, a patient, after enlarging on the peculiar difficulties of the case, asserts, (and which, by the by, are in italics) that the tooth was removed "without any bleeding whatever;" had the testimonial containing this passage not been appended to a scientific work, it would have been passed unnoticed by me; but, the extraction of a molar, firmly fixed and affording little substance for the grasp of the forceps, ("without any bleeding whatever,") is a feat partaking so much of the marvellous, we are led to regret that the circumstance was not made the subject of special comment.

It is most probable, sir, you would not have been troubled with this communication had not the interest I take in matters connected with odontology, led me to inspect the invention for myself, and after the very excellent description with which Mr. Gilbert favored me, I am enabled to assert that the end for which he constructed the apparatus is yet unattained. In the first place, the fulcrum is not external to the mouth, because it is allowed to rest on the incisor teeth. Mr. Gilbert assured me the pressure was slight and gave rise to no ill-effects, but any amount of pressure must be objectionable; the indentation in the material covering the fulcrum show that pressure *does exist.* Secondly, the "painful wriggle of the forceps," is still required; in other words, Mr. Gilbert employs the *alternate lateral motion;* for the purpose, as he explained, of detaching the tooth from its connection with the socket, (referring to Robinson,) I find his directions for the application of the forceps comprise the same words—the only difference in the two operators, as far as I can discern, consists in one trusting to his wrist to detach and elevate the tooth, while the other, by the employment of a fulcrum, converts his forceps into a lever for the same purpose, the alternate movements being used by either. Lastly, if the key is ever requisite under any circumstances, Mr. Gilbert's invention cannot supply its place. Robinson limits its application to those cases where one side of the tooth is so far removed as to leave no substance for the grasp of the forceps—as Mr. Gilbert uses this instrument, of course he can only operate in those instances where sufficient substance is left for him to grasp, as the use of a fulcrum certainly does not remove the necessity of securing the member firmly prior to attempting its extraction.

I forbear to trespass further on your attention, save to repeat my conviction that, although Mr. Gilbert deserves all credit for the ingenuity he has evinced in his laudable endeavors to alleviate human suffering, he has not gained the object he intended—that whether John Hunter be right or wrong, the fulcrum apparatus does not go far to strengthen his position.

GEORGE BROWN.

Lectr. Vety. Medl. Royal Agrictr. College, Cireucester.

*J nuary*, 1851.

